# Lutein Supplementation for Eye Diseases

**DOI:** 10.3390/nu12061721

**Published:** 2020-06-09

**Authors:** Long Hin Li, Jetty Chung-Yung Lee, Ho Hang Leung, Wai Ching Lam, Zhongjie Fu, Amy Cheuk Yin Lo

**Affiliations:** 1 Department of Ophthalmology, LKS Faculty of Medicine, The University of Hong Kong, Hong Kong, China; u3548400@connect.hku.hk (L.H.L.); waichlam@hku.hk (W.C.L.); 2 School of Biological Sciences, Faculty of Science, The University of Hong Kong, Hong Kong, China; jettylee@hku.hk (J.C.-Y.L.); lhhkoala@connect.hku.hk (H.H.L.); 3 Department of Ophthalmology, Boston Children’s Hospital/Harvard Medical School, Boston, MA 02115, USA; 4 Manton Center for Orphan Disease, Boston Children’s Hospital, Harvard Medical School, Boston, MA 02115, USA

**Keywords:** age-related macular degeneration, antioxidant, carotenoid, diabetic retinopathy, myopia, cataract, nutrition, retina, retinopathy of prematurity, xanthophyll

## Abstract

Lutein is one of the few xanthophyll carotenoids that is found in high concentration in the macula of human retina. As *de novo* synthesis of lutein within the human body is impossible, lutein can only be obtained from diet. It is a natural substance abundant in egg yolk and dark green leafy vegetables. Many basic and clinical studies have reported lutein’s anti-oxidative and anti-inflammatory properties in the eye, suggesting its beneficial effects on protection and alleviation of ocular diseases such as age-related macular degeneration, diabetic retinopathy, retinopathy of prematurity, myopia, and cataract. Most importantly, lutein is categorized as Generally Regarded as Safe (GRAS), posing minimal side-effects upon long term consumption. In this review, we will discuss the chemical structure and properties of lutein as well as its application and safety as a nutritional supplement. Finally, the effects of lutein consumption on the aforementioned eye diseases will be reviewed.

## 1. Introduction

Although there are about 850 types of carotenoids discovered and characterized in nature to date [1], very few of them are present in human tissues. Among them, lutein and its stereoisomers zeaxanthin and *meso*-zeaxanthin, are the only carotenoids present in the human retina [2]. They belong to the carotenoid class named xanthophyll, which contains oxygen and is less hydrophobic compared with the other class named carotene, which is purely hydrocarbon and more hydrophobic [3].

### 1.1. Dietary Lutein and Its Uptake

Carotenoids are mostly synthesized in plants and microorganisms, but not in human. Hence, lutein must be obtained from the diet. Green leafy vegetables such as kale, spinach, broccoli, peas, and lettuce as well as egg yolk are the most common sources of xanthophylls, including lutein and zeaxanthin [4]. They are also found in einkorn, Khorasan, durum wheat and corn related food products (Table 1).

Interestingly, the distribution of lutein and zeaxanthin varies in different food types. The ratio of lutein/zeaxanthin in green vegetables is reported to be 12 to 63 (for instance, kale has the highest ratio) while in orange-yellow fruits and vegetables, the ratio is 0.1 to 1.4 only [6]. A small amount of lutein and zeaxanthin is also observed in breads made with modern wheat variety. Compared with plant-based foods, egg yolks are considered to be a better source of lutein and zeaxanthin because the high fat content of eggs increases the bioavailability of carotenoids [7]. Carotenoid content in egg yolk largely depends on the type of feed given to the hen, which contains the esterified forms of lutein and zeaxanthin along with small amounts of lycopene and β-carotene [8].

Prior to exerting their nutritional effects, carotenoids must first be absorbed and transported into circulation. Thereby, a thorough understanding on the release, absorption, transport, and accumulation of carotenoids in the eye is critical to assess its health benefits. Carotenoids are hydrophobic in general, which means they are soluble in fats but not in the aqueous medium of human digestive system. However, due to the presence of hydroxyl group, lutein and zeaxanthin are relatively polar compounds compared with hydrocarbon carotenoids such as β-carotene and lycopene.

The bioavailability of lutein and zeaxanthin in ocular tissue depends on their absorption from food [9,10,11], which is affected by several factors including:

- (i) the nature of the food matrix (natural form or supplementation);
- (ii) the amount and nature of dietary fat, which promote the circulation of carotenoids;
- (iii) presence of phospholipids;
- (iv) presence of dietary fibers;
- (v) properties of dietary carotenoids.

Bioavailability of carotenoids is greatly influenced by the conditions of food matrices [10], where the release rate of carotenoids from food matrix and subsequent absorption are important decisive factors in providing the desired health benefits. It has been observed that the release of lutein, zeaxanthin and β-cryptoxanthin from fruits (orange, kiwi, grapefruit and sweet potato) is almost complete, while those from green vegetables (spinach and broccoli) are 19–38% only [12]. However, carotenoids present in the food matrix can be released prior to consumption by food processing and heat treatment, and thus enhance the absorption rate [13]. 

After consumption, dietary carotenoids are dispersed in gastric juice and incorporated into lipid droplets, which subsequently are transferred into mixed micelles containing bile salts, bile phospholipids and dietary lipids. Following the uptake in the intestinal cells through simple diffusion, micelle absorption and receptor-mediated active transports, the dissolved carotenoids enter the bloodstream for transportation [14]. Since the highest concentration (i.e., dissolution) of carotenoids in micelles corresponds to greater absorption and transport to plasma, high fat diets generally facilitate the absorption of dietary carotenoids by promoting the generation of intestinal micelles.

Typically, circulating carotenoids are transported primarily through low-density lipoprotein (LDL; 55%), high-density lipoprotein (HDL; 33%) and very low-density lipoprotein (VLDL; 10–19%) [15]. On the contrary, the distribution of lutein and zeaxanthin in LDL and HDL are almost equal, but more favorable in HDL [16]. Recently, a cross-sectional study has observed a significant positive correlation between lipoprotein concentrations and serum levels of lutein and zeaxanthin and concluded that altering lipoprotein concentrations may affect the levels of retinal lutein and zeaxanthin [17].

### 1.2. Distribution of Lutein in the Human Body

The distribution of lutein among different human tissues is not uniform, with the highest amount concentrated in the macula. Macula lies in the central retina, in the posterior part of the eye and is responsible for visual acuity and central vision due to its high concentration of photoreceptor cells. In the macula, there are two other types of carotenoids, namely, zeaxanthin (a stereoisomer of lutein that is also obtained from the diet) and *meso*-zeaxanthin (a metabolite of lutein that is formed at the macula via metabolic transformation) [18]. The three carotenoids exhibit regional dominance in the macula, with lutein present at the highest quantity in the periphery, zeaxanthin in the mid-periphery, and *meso*-zeaxanthin in the epicenter, respectively [19]. Together, they form the retinal macular pigment, which is essential for maintaining optimal visual performance and is often used as a proxy for predicting the risk of developing macular diseases [20]. Using confocal resonance Raman microscopy, a more recent study further mapped the spatial distribution of lutein and zeaxanthin in the human retina. It provided new data and discovered that zeaxanthin level is in fact the highest in the fovea and drops sharply in the peripheral macula. Lutein, on the other hand, has a more even distribution across the macula at a lower concentration in the macula compared with zeaxanthin [21]. Lutein is also found in the human lens, protecting it against age-related eye diseases such as cataract [22]. Moreover, some studies indicated that lutein, due to its hydrophobic nature, is also distributed in adipose tissue, leading to a reduction in retinal lutein level in obese people hence predisposing them to various eye diseases [23].

Another characteristic of lutein is that it can be retained in the human retina for a sustained period of time. When subjects received dietary supplementation of lutein, both their serum lutein concentration and retinal lutein level, as measured by macular pigment optical density (MPOD), increased. Around 50 days to 3 months after discontinuation of lutein supplementation, lutein serum concentration returned to baseline but MPOD remained elevated [24,25]. Multiple explanations have been proposed. Some researchers argued that the xanthophyll cleavage enzyme, β,β-carotene-9’,10’-dioxygenase, in human is much weaker than that in rodents. Hence, the capture and cleavage of lutein and other carotenoids in human are more ineffective, resulting in a high level of carotenoids that are accumulated and retained in the human retina [26]. Another explanation would be the involvement of the adipose tissue. Some scientists proposed that lutein stored in the adipose tissue may be supplied to the retina to form macular pigment, since a negative correlation between changes in adipose tissue lutein concentration and changes in MPOD was observed after cessation of lutein supplementation [27].

### 1.3. Chemical Structures and Properties of Lutein

**Figure 1:**
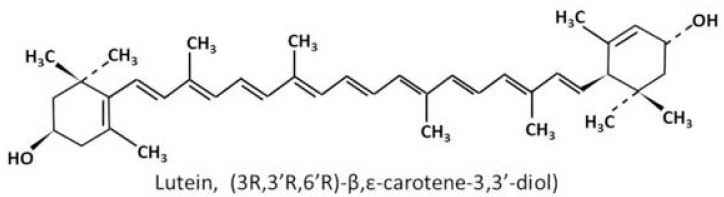
Chemical structure of lutein [29].

The biochemistry and metabolism of lutein have been reviewed in detail recently [28]. Briefly, lutein is a carotenoid. Carotenoids have a 40-carbon skeleton formed by two 20-carbon precursors, geranylgeranyl pyrophosphate (GGPP), joining in a head-to-head manner. The long carbon skeleton carries conjugated double bonds with linear and cyclic alternatives, which allows structural diversities, i.e., *cis* or *trans* configuration. Carotenoids consist of purely hydrocarbons are classified into the subgroup named carotenes, while those with at least one oxygen atom in their polyene chain are grouped as xanthophyll. Lutein belongs to the latter subgroup, with two hydroxyl groups attached to the terminal ionone rings on both sides (Figure 1).

Owing to its chemical structure, lutein possesses various properties that helps the maintenance of retinal function and prevention of a number of eye diseases. The two hydroxyl groups in lutein make it more polar and hydrophilic compared with carotene, allowing itself to better react with oxygen in the serum and act as an anti-oxidant by effectively scavenging reactive oxygen species (ROS) [30] including superoxide anion (O_2_^−^), perhydroxyl radical (HO_2_·), and hydroxyl radical (OH·). Radicals contain unpaired electrons in their outer shell; hence, they are highly chemically unstable and tend to react with other molecules via further reduction or oxidation in order to reach a stable state. Such actions could damage the cell membrane lipid bilayer, proteins and DNA, and impair normal functioning of mitochondria, ultimately leading to cell necrosis [31]. By reacting with these ROS, lutein can effectively reduce cellular damage caused by radical actions. During the process, ROS accepts the missing electrons from lutein, thus unable to further oxidize other cellular structures. Moreover, superoxide radicals can be converted into hydrogen peroxide and singlet oxygen by non-enzymatic reactions. Singlet oxygen, similar to other free radicals, can also exert cellular damage through oxidative reactions. Lutein can prevent such damage by quenching singlet oxygen. During the process, the energy of singlet oxygen is transferred to lutein, which then dissipates the energy without undergoing physical changes and hence can be reused in another quenching cycle.

Due to the orientation of the liposomal membrane, lutein is found to be the best filter of blue light with the highest efficacy compared with other carotenoids [32]. Our eyes are constantly exposed to potentially toxic blue light, with sources from smartphones, computers, digital tablets, and LED (Light Emitting Diodes) lamps for interior lighting [33]. Blue light, with a typical wave length ranging from 450 to 495 nm, is of high energy, hence can prompt free radical formation and induce oxidative stress to the eyes, resulting in increased risk of macular diseases and cataract [34]. As the peak wavelength of lutein’s absorption is around 460 nm which lies within the range of blue light, lutein can effectively reduce light-induced damage by absorbing 40% to 90% of incident blue light depending on its concentration [35]. The outer plexiform layer of the fovea, where the majority of axons of rod and cone photoreceptor cells are located, is the retinal layer having the highest density of macular carotenoids including lutein [36]. Hence the photoreceptors are protected against photo-oxidative damages from blue light.

Furthermore, lutein is shown to possess anti-inflammatory properties. In vitro studies indicated that lutein can inhibit pro-inflammatory molecules such as cyclooxygenase-2 (COX-2), inducible nitric oxide synthase (iNOS), and nuclear factor-kappa B (NF-κB) [37]. Moreover, animal studies showed that the levels of these molecules, together with interleukin 1β (IL-1β) in lutein-treated mice after ischemia/reperfusion injury, were significantly decreased when compared with that of the vehicle-treated group [38]. It is suggested that lutein helps to prevent the increase in oxidation-induced cytokines and the upregulation of inflammation-related gene expression [39], as well as decreases complement factor D, which is a rate-limiting enzyme involved in the alternative complement activation pathway [40]. Besides, lutein is able to reduce VEGF expression, which is vital in inducing unregulated angiogenesis under pathological conditions [41]. As inflammation and abnormal angiogenesis in retinal vasculature are major pathogenic mechanisms of many ocular diseases, lutein’s functions in suppressing inflammatory response and VEGF expression make it effective in reducing the severity of these diseases.

There is also evidence demonstrating other functions of lutein, including its ability to improve visual acuity and contrast sensitivity [42], as well as its neuroprotective effect in reducing cell loss and cell apoptosis after retinal ischemia/reperfusion injury [43]. Lutein could attenuate apoptotic and autophagic responses after hypoxic insult by improving glial cell viability as well as suppressing autophagosome formation [44]. Lutein is also important for cell-cell communication through intercellular gap junctions due to its chemical structure [45]. Moreover, as the levels of lutein in the retina and the visual cortex are found to be significantly similar in human [46], it is suggested that lutein plays a substantial role in converting photic signals into electrical impulses in the retina, as well as neural transmission to the visual cortex.

Nonetheless, it is worth noting that although promising results from a number of in vitro and animal studies indicated multiple beneficial roles of lutein in retinal health, evidence concerning the ability of lutein to carry out these functions in human remains to be established.

## 2. Safety Profile of Lutein as Eye Health Supplement

While there is a lack of univocal data on the optimal dosage of lutein supplementation, it has a relatively high safety profile as indicated by many studies and is categorized as Generally Regarded as Safe (GRAS) by the US Food and Drug Administration (FDA) [47]. German and Canadian studies reported an average adult’s lutein consumption of 1.9 mg and 1.4 mg per day, respectively [48], whereas Americans consume around 1–2 mg of lutein and zeaxanthin on average per day [49]. However, these consumption values are significantly below the dosage tested by the Age-Related Eye Disease Study 2 (AREDS2) randomized clinical trial published in 2013, which reported no adverse health effects except minor skin yellowing with a daily lutein dose of 10 mg per day persisted for 5 years among over 4000 patients with age-related macular degeneration [50]. In fact, according to the Council for Responsible Nutrition (CRN), lutein is safe at an intake up to 20 mg/day [51]. Nonetheless, studies have tried even higher lutein levels without self-reported side effects. Two other clinical studies investigating the effects of lutein on retinitis pigmentosa and MPOD have used lutein doses at 40 mg/day for 9 weeks, 20 mg/day thereafter up to 26 weeks and 30 mg/day for 120 days, respectively. No adverse health effects were reported [52,53]. Moreover, an animal study indicated no signs of toxicity upon lutein administration of 639 mg/kg/day in rats [54]. However, it should be acknowledged that experimental rodents are usually given drug dosages at least 10 times higher than the human dose clinically; therefore, it is inappropriate to replicate this study in clinical investigations using such a high concentration of lutein. According to the formula used in converting drug doses between species (conversion factor for human dose to rat dose = 6.2) [55], 40 mg/day dose in a normal 60 kg adult is equivalent to a daily dose of 4.1 mg/kg in rat (6.2 × 40 mg/60 kg). This seems definitely minimal comparing with 639 mg/kg/day used in the aforementioned rat study [54]. The absence of observable lutein toxicity in human and rat after lutein supplementation provided a strong evidence for the high safety margin of lutein. 

The side effects of excessive lutein consumption seem to be mild and rarely reported. There is one case study reporting bilateral “foveal sparkle” in an elderly Asian woman who consumed an exceptionally high lutein level with daily lutein supplement at 20 mg and a high intake of dietary lutein (a broccoli, kale, spinach, and avocado smoothie every morning) for a duration of 8 years. Her serum lutein level measured by HPLC (519 ng/mL) was 2.9 times higher than the un-supplemented population (182 ± 196 ng/mL). Crystals in the inner layers of the foveal region within her right eye eventually dissolved 7 months after discontinuation of lutein, while the crystal in her left eye persisted [56]. 

Although further studies investigating the effects of long term consumption of lutein supplements are needed, people’s average lutein intake, as indicated by various population-based surveys, is still considerably below the tested dosages in the aforementioned studies (e.g., 10 mg in AREDS2 [50] and 20 mg/day in CRN [51]). In fact, some studies have highlighted the important role of regular dietary supplementation of antioxidants like lutein in maintaining general eye health [57], such as promoting visual acuity and contrast sensitivity while reducing glare disability and discomfort, on top of receiving drug and surgical treatments targeting for specific eye diseases. Hence, it is reasonable to recommend consumption of adequate amount of lutein due to its beneficial effects.

## 3. Lutein Supplementation and Age-Related Macular Degeneration

### 3.1. Age-Related Macular Degeneration: Background

Located in central retina, the macula possesses high concentration of photoreceptors which give rise to high-resolution visual acuity and sharp central vision. However, with age, the macula is susceptible to gradual degenerative changes, which results in one of the most common aging eye diseases called age-related macular degeneration (AMD). Due to the longer life expectancy and therefore a rising aging population, AMD is now one of the leading causes of blindness and irreversible visual impairment. It is estimated that the number of AMD patients worldwide would grow from 196 million in 2020 to 288 million by 2050 [58]. 

Based on clinical presentations and ophthalmological examinations, there are a number of classification systems categorizing the stages and severity of AMD. Below are the two commonly used ones (Table 2, Table 3).

Drusen are round, yellowish deposits found in both the macular and peripheral retina, consisting mostly of protein, esterified and unesterified cholesterol [62]. Their sizes can be clinically observed under the color fundus photography to assess the patient’s AMD stage.

At early stages of AMD, patients are usually asymptomatic or carry mild symptoms such as mild central distortion in vision and having difficulty to read under dim environment [60]. Diagnosis is normally made based on drusen discovery upon routine ophthalmological examination. Advanced stages of AMD present symptoms like distorted vision and formation of a dark patch called scotoma which occludes central vision [60]. According to its pathophysiology, it can be subdivided into neovascular AMD and atrophic AMD.

Neovascular AMD progresses rapidly within weeks or months. It involves neovascularization below the retinal pigment epithelium (RPE), or above the RPE within the subretinal space, or within the retinal circulation forming anastomosis with the choroidal circulation [63]. Retinal hemorrhage occurs subsequently when the weak walls of the newly formed vessels from angiogenesis break, followed by exudate accumulation and subsequent formation of fibrous scar tissue, leading to severe visual impairment. On the other hand, atrophic AMD has a much slower progression characterized by the gradual degeneration of cells in the macula and the RPE layer, forming a geographic atrophy region where the size and border can be assessed under fundus autofluorescence imaging and spectral-domain optical coherence tomography [60]. As the atrophy region expands slowly, it usually takes years or even decades for the patient’s vision to decline.

AMD is a multifactorial disease with both environmental and genetic contribution. Major risk factors include age, gender, ethnicity, family background, and smoking. 

### 3.2. Age-Related Macular Degeneration: Pathogenesis and Current Treatments

There are many possible physiological mechanisms leading to AMD development, with the major pathways being exposure to oxidative stress and inflammatory reactions.

The retina is highly susceptible to ROS. It is the major site of signal transduction where light photons are converted into electrochemical signals, predisposing it to photo-oxidative stress. Moreover, the rod cells within the retina contains a large number of mitochondria, which are the major site of ROS generation. During oxidative metabolism in mitochondria, electron transport chain is needed for ATP synthesis. Electrons, which undergo sequential reduction and oxidation reactions in the process, may leak out and form ROS. Excess ROS can exert oxidative damages which lead to cytotoxic effects such as point mutations of mitochondrial DNA and early cell senescence by shortening of telomeric DNA [64,65]. 

However, the most important mechanism by which ROS mediates the development of AMD is attributable to lipid peroxidation of polyunsaturated fatty acids. The outer segment of photoreceptor cells in the retina is found to contain a high level of polyunsaturated fatty acids. These acids are particularly susceptible to ROS oxidation, producing reactive aldehyde intermediates, which possess cytotoxic effects [66]. Normally, the polyunsaturated fatty acids constantly shed off from the photoreceptor cells and are phagocytosed by the neighboring RPE cells. However, with age, the RPE cells become inefficient in phagocytosis, leaving behind a large amount of oxidized proteins and undigested intermediates within the cytoplasm in form of lysosomal residual bodies known as lipofuscin [67]. It is estimated that the percentage by volume of lipofuscin in RPE cell cytoplasm increases by 19% from childhood to the age of 80 [68]. These lipofuscin granules not only inhibit the anti-oxidant ability of RPE cells but also suppress their regular physiological functions [69]. Without the metabolic support provided by the RPE cells, photoreceptor cells begin to degenerate and die, contributing to the visual impairment in patients with late AMD.

Inflammatory reactions are mainly mediated by the formation of drusen between RPE and Bruch’s membrane, which is the innermost layer of choroid. Some studies indicated that drusen formation is due to the exudative accumulation of extracellular wastes subsequently leading to thickening and permeability changes of Bruch’s basement membrane as well as the breakdown of choriocapillaries [70,71]. Drusen contain proteolytic fragments and cell debris resulted from RPE cell degeneration, triggering complement activation and hence local inflammation [72]. 

To date, there are a few treatment options for neovascular AMD but each with considerable limitations. Ever since anti-VEGF compounds were proven able to inhibit abnormal growth of blood vessels and hence stabilize vision, anti-VEGF drugs (e.g., bevacizumab, ranibizumab, and aflibercept) by intravitreal injection have been used as a standard treatment. Although most AMD patients’ vision remained stable and around 40% of them experienced visual improvement after anti-VEGF treatment [29], the need for regular injection and the high cost of injection make such treatment option relatively inconvenient. In addition, more than 50% AMD patients displayed poor responses to anti-VEGF agents [73]. Choroidal neovascularization (CNV) can also be treated by thermal laser photocoagulation and photodynamic therapy. Thermal laser photocoagulation is reserved for the non-center involved CNV because of the destructive nature of thermal laser burn. Photodynamic therapy selectively damages the neovascular lesion while preserving neighboring tissues. However, recurrence rate is high, and there is still lack of evidence that this procedure can provide significant vision improvement.

There are no approved drugs that are proven to be efficient in treating atrophic AMD, and currently the main strategy is treatment with antioxidants, multivitamins, and minerals (AREDS2 formula), as well as to reduce risk factors that may increase occurrence and progression of atrophic lesions. As a number of animal and clinical studies indicated lutein’s therapeutic effects in alleviating oxidative and inflammatory damages, which are two major pathological processes in AMD, clinical trials focusing on the association between lutein intake and AMD development have been performed in an attempt to devise a novel strategy for AMD treatment and prevention with higher efficacy and lower adverse health effects.

### 3.3. Lutein and AMD (Clinical Studies)

Age-Related Eye Disease Study (AREDS) is one of the most important large scale clinical studies investigating the association between different nutrients intake and AMD progression. The initial AREDS study published in 2000 revealed that a combination of nutrient supplementation (500 mg vitamin C, 400 IU vitamin E, 2 mg cupric oxide, 80 mg zinc and 15 mg β-carotene) taken orally every day can effectively reduce the risk of progression to late AMD by 25% [61]. However, since it was later discovered that β-carotene may increase the risk of lung cancer among cigarette smokers, AREDS2 published in 2013 modified its formula by removing β-carotene while adding lutein and zeaxanthin. Although the risk of late AMD progression was not further reduced compared with the previous formula, removal of β-carotene and the addition of lutein and zeaxanthin exerted similar protective effects without imposing additional risks to smokers [50]. Other case-control studies also supported the beneficial effects of lutein intake in AMD. It is estimated that dietary lutein intake is strongly associated with the reduced risk of different kinds of AMD, with an odds ratio of 0.65 for neovascular AMD, 0.45 for atrophic AMD and 0.73 for large or extensive intermediate drusen [74]. Moreover, the Blue Mountains Eye Study analyzed the dieting habit of over 2000 Australians and followed them up to investigate the incidence of AMD. The results indicated that individuals with the highest lutein and zeaxanthin consumption had a 65% reduction in neovascular AMD compared with those with the lowest intake [75].

Besides looking into the intake of lutein supplements, plasma level of lutein and retinal macular pigment level are two other factors shown to be predictors of AMD risks. Early studies had suggested the possibility that higher blood levels of antioxidants, especially carotenoids such as lutein, may be associated with AMD risk reduction [76]. Later analysis of prospective population studies further illustrated that a high plasma level of lutein and zeaxanthin (>0.56 μM) could achieve a 79% risk reduction of age-related maculopathy comparing to a low level (<0.25 μM) [77]. Concerning macular pigment level, a case-control study comparing retinas from donors with and without AMD indicated that donors having the highest quartile of macular pigment level had an 82% risk reduction of AMD compared with the lowest quartile [20]. Several studies also looked at MPOD. Lutein Antioxidant Supplementation Trial II (LASTII) and Lutein Intervention Study Austria (LISA) showed that MPOD increased upon lutein supplement but decreased without supplementation. The increase is most prominent among subjects with low baseline MPOD [78,79]. The Combination of Lutein Effects in the Aging Retina (CLEAR) study also reported similar results, and further pointed out that a high MPOD retarded visual acuity reduction during aging [80].

On the other hand, a number of studies failed to show a positive association between lutein intake and AMD. Two prospective follow-up studies tracking the diet patterns of over 110,000 men and women showed no evidence of lutein’s protective effects against early AMD [81]. The Beaver Dam Study following 1709 Americans for 5 years also revealed a lack of association [82], while the Carotenoids in Age-related Eye Disease Study (CAREDS) showed that the risk reduction of lutein against intermediate AMD was only statistically significant among women older than 75 years old [83]. A summary of clinical studies related to lutein supplementation and AMD is listed below (Table 4 and Table 5).

The inconsistent findings of these studies may be attributable to several reasons. Firstly, even with the same lutein dose administered, the rate and extent of lutein absorption and tissue uptake may be different among subjects with different race, gender, and age. Secondly, for some small-scale prospective studies, the incidence of AMD cases may be too few to establish a significant association. Thirdly, some studies investigating early AMD incidence required self-reporting. Since symptoms of early AMD are not apparent, it may result in inaccuracies in reporting and misclassification of cases.

### 3.4. Lutein and AMD (Experimental Studies)

A number of animal studies have used apolipoprotein E-deficient mice (apoE^−/−^) to mimic the pathological changes of AMD in human. This genetic mouse model develops spontaneous hypercholesterolemia in the first few weeks of life and subsequently develops RPE morphological alterations similar to AMD, such as formation of cytoplasm vacuoles in RPE, swelling and thickening of Bruch’s membrane, etc. By supplementing a complex of lutein, multivitamins and glutathione to apoE^−/−^ mice, these structural changes can be delayed or even reversed, and VEGF expression can be reduced [41,88]. Moreover, lipofuscin accumulation was reduced, and photoreceptors were preserved in the mouse retina when treated with the AREDS2 formula consisting of lutein and zeaxanthin [89]. In an in vitro study using cultured retinal pigment epithelial cells (ARPE-19) that were exposed to H_2_O_2_, which induced increased ROS formation and apoptosis as well as reduced cell viability, administration of lutein was able to reverse these pathological damages in a concentration-dependent manner [90]. However, limitations do exist when using rodents as animal models in AMD research. Notably, rodents do not have macula, which is primate-specific. Moreover, carotenoids are not accumulated in the rodent retina. Hence, the effect of lutein supplementation on MPOD cannot be studied in rodent models.

In view of all these studies, the association between lutein and AMD risk reduction is generally accepted due to lutein’s anti-oxidative and anti-inflammatory properties. However, its long-term protective effects and optimal doses for treating different stages of AMD remain to be investigated. Besides putting emphasis on lutein supplementation, patients are also advised to pay attention to other risk factors such as smoking and vascular diseases, which may affect AMD incidence and progression as well.

## 4. Lutein Supplementation and Diabetic Retinopathy

### 4.1. Diabetic Retinopathy: Background

Diabetic retinopathy (DR) is one of the most common microvascular complications of diabetes, affecting around one third of patients with diabetes [91]. It remains the leading cause of preventable blindness among working adults, and it is estimated that 1 out of 39 blind people suffered from blindness because of DR [92]. With an alarming surge of prevalence by 64% from 2000 to 2010 [92], DR is mostly concentrated in high income regions such as in Western Europe and North America. However, DR prevalence is also gradually increasing in many Asian countries such as China and India due to economic growth and lifestyle changes.

DR is mainly classified into two pathological stages, the non-proliferative and proliferative stage. Non-proliferative diabetic retinopathy (NPDR) is the early stage of DR, characterized by intraretinal hemorrhages, microaneurysms and vasculature damages leading to exudate accumulation. The pathological alterations are usually mild and gradual, which makes NPDR patients asymptomatic or gives them mild visual impairment. Subsequently, nonperfusion of retinal vasculature may develop, blocking nutrients and oxygen delivery to retinal neural cells, which are constantly at a state of high metabolic demand. This eventually leads to neuronal damage due to hypoxia, forming fluffy white patches on the retina known as cotton wool spots [93]. The aberrant formation of new but fragile blood vessels as a response to continuous ischemia marks the progression into the advanced stage of DR, proliferative diabetic retinopathy (PDR). Neovascularization in retina can lead to vitreous hemorrhage or tractional retinal detachment, which severely impair vision. Diabetic macular edema is also another pathological manifestation of DR which can occur during both NPDR and PDR. The breakdown of blood-retinal barrier leads to swelling within the macula and progressive vision loss.

There are many classification scales to categorize DR according to its progression or severity (Table 6). The International Clinical Disease Severity Scale for DR is a simplified scale used by many clinicians, which is based on the Wisconsin Epidemiologic Study of Diabetic Retinopathy (WESDR) and the Early Treatment of Diabetic Retinopathy Study (ETDRS) [94].

### 4.2. Diabetic Retinopathy: Pathogenesis and Current Treatments

DR is a multifactorial disease with a complex pathogenic process. One of the key risk factors of DR is elevated blood glucose level. Clinical studies indicated that intensive glycemic control is vital in preventing or delaying progression of DR. High level of hemoglobin A1c (HbA1c), a marker for hyperglycemic condition, is associated with accelerated progression from NPDR to PDR [96,97]. This is attributable to the formation and accumulation of advanced glycation end products (AGEs) when proteins are exposed to long-standing hyperglycemic environment [98]. Production of AGEs generates ROS as by-products, exerting oxidative stress to the retina. This leads to retinal mitochondrial dysfunction, DNA damages and capillary cells apoptosis, resulting in retinopathy [99]. 

Laser intervention and anti-VEGF treatment are two main management strategies currently used to treat DR in the clinical setting. Studies revealed that laser therapy is successful in reducing the rate of vision loss by half within 3 years, and the results are most prominent when diabetic macula edema is present [100]. However, since the process is invasive in nature, there are risks of adverse effects such as foveal burn, formation and spread of laser scars, and retinal fibrosis [101]. Other ocular functions such as visual acuity and dark light adaptation may also be affected. VEGF plays an important role in increasing vascular permeability and promoting angiogenesis in PDR; therefore, anti-VEGF therapies are developed to stop its progression. Similar to neovascular AMD, anti-VEGF drugs can be administered through intravitreal injection for patients with macula edema or diabetic retinopathy. Although anti-VEGF therapies are generally regarded as effective and well tolerated, the costs are high, and occasional side effects are reported, such as persistent hypertension, transient formation of floaters, and increase in intraocular pressure [102].

Compared with these treatments, lutein supplementation appears to be a natural and safe alternative with lower cost and less invasiveness. Its effects on DR have been widely investigated in clinical and animal studies.

### 4.3. Lutein and Diabetic Retinopathy (Clinical Studies)

Among type 2 diabetic patients, a retrospective study showed that supplementation of lutein and zeaxanthin can improve retinal thickness and function as measured by optical coherence tomography (OCT) and multifocal electroretinography (mfERG), indicating the protective effects of carotenoids on visual function in the diabetic state [103]. The number of study investigating lutein and DR is relatively limited, with the main emphasis on studying the effects of lutein and zeaxanthin level in retina (represented by MPOD) or in blood during DR development. The results were consistent with the general idea of lutein’s beneficial effects, showing that a higher lutein plasma level or a higher MPOD is associated with a lower risk of DR development or progression. The studies are summarized by the two tables below (Table 7 and Table 8).

### 4.4. Lutein and Diabetic Retinopathy (Animal Studies)

Animal studies of diabetic retinopathy (DR) mainly used alloxan- or streptozotoxin-induced diabetic mice model. Alloxan and streptozotoxin are taken up by pancreatic beta cells through GLUT2 glucose transporter, subsequently leading to toxicity and necrosis. This inhibits insulin production by pancreatic beta cells and hence is able to mimic a hyperglycemic state similar to that of insulin-dependent DM in human [108]. In these mice, multiple pathological changes are observed, such as increased oxidative stress, impaired visual function, inflammatory activation, and morphological alteration. Studies indicated that these changes are significantly restored after lutein supplementation. Firstly, lutein reduces oxidative stress marked by a decrease in extracellular signal-regulated kinase (ERK) activation and brain-derived neurotrophic factor (BDNF), as well as an increase in glutathione (GSH) and glutathione peroxidase (GPx) [109,110]. Secondly, lutein improves visual function by restoring amplitude of b-wave on electroretinogram (ERG) produced by depolarization of ON-bipolar cells in inner retina [110,111]. Thirdly, lutein and zeaxanthin supplement can reduce the level of inflammatory mediators that are responsible for causing neovascularization in DR, such as VEGF, NF-κB and IL-1β [112]. Lastly, many morphological damages in the diabetic retina, including thinning of inner nuclear layer, inner plexiform layer, and ganglion cell layer, as well as deprivation of pigment granules and mitochondria in RPE, can be alleviated upon lutein administration [109,113].

Another study used streptozotoxin-induced diabetic rats as animal model for DR with lutein as one of the nutritional supplements, and it showed equally promising results [112]. Diabetic rats demonstrated a 3–4 fold increase in retinal capillary degeneration and cell apoptosis, as well as a 25% reduction in ERG a- and b- wave amplitude compared to normal rats. However, these morphological and functional impairments were significantly ameliorated upon treatment with nutritional supplements.

It is important to note that the dosages of lutein used in these animal studies were usually much higher than that used in clinical trials, which made its protective effects more prominent. Hence, in human trials or clinical prescriptions, it is necessary to strike a balance between elevating lutein dosage to reproduce similar protective effects obtained in animals and observing the safety limits of lutein in human.

## 5. Lutein Supplementation and Retinopathy of Prematurity

### 5.1. Retinopathy of Prematurity: Background

Retinopathy of prematurity (ROP) is a disorder of developing retinal blood vessels in premature newborn infants. In the United States, ROP is the second leading cause of childhood blindness, making 400 to 600 infants legally blind each year [114]. Globally, it is estimated that over 180,000 newborns develop ROP in a year, 20,000 of whom suffer from severe visual impairment or even blindness [115]. Long term visual complications such as myopia and macular scarring resulting in reduced central visual acuity may occur later in life in ROP patients, compromising their quality of life.

Birth weight and gestational age are the two greatest risk factors of ROP, with a lower weight and smaller age associated with increased ROP risk. It is estimated that around 66% of newborn with birth weight ≤1250 g suffer from ROP, and the proportion rises to 82% among those weighing ≤1000 g [116]. Meanwhile, in a study of ROP among discordant twins in China, the mean gestational age of newborn babies having ROP was 28.56 weeks, which was significantly lower than those without ROP (31.35 weeks). The mean gestational age of those having severe ROP was even lower at 27.75 weeks [117], as the underdeveloped retinal vasculature in premature infants is more susceptible to ROP pathological changes. Other known risk factors include the postnatal use of oxygen supplementation, low levels of insulin-like growth factor-1 (IGF-1), intraventricular hemorrhage, blood transfusion, and sepsis [118].

### 5.2. Retinopathy of Prematurity: Pathogenesis and Current Treatments 

Owing to the relatively hypoxic intrauterine environment during pregnancy, production of VEGF is stimulated, initiating development of retinal vasculature, which begins at around 16th week of gestation and continues until term when the adult pattern is reached [119]. When the infant is born prematurely, the incomplete retinal vessel development makes the retina susceptible to ischemic and oxidative stress, predisposing the retinal vasculature to pathological hypoxic changes that lead to ROP.

ROP is commonly classified into two phases. The first phase (22–30 weeks after conception) begins right after the birth of the preterm infant as it starts to breathe. Compared to the intrauterine environment, the immature retina is exposed to a relatively hyperoxic environment after birth under oxygen supplementation, which leads to the down regulation of VEGF and IGF-1. Under a low level of angiogenic factors, the normal development of retinal vasculature is interrupted, and the developed vessels regress [118]. Such interruption further exaggerates the metabolic imbalance in which the low vessel density of the underdeveloped vasculature cannot meet the retinal oxygen demands, marking the progression into the second phase (31–34 weeks after conception). The now hypoxic retina triggers overproduction of VEGF and IGF-1, leading to aberrant formation of disorganized retinal blood vessels by endothelial cell differentiation and migration, a process known as neovascularization. Gradually, the abnormal vessels form ridges and sprout into the vitreous spaces. Total tractional retinal detachment may occur in the last stage of ROP due to cicatricial contraction of aberrant vessels, which eventually result in severely impaired vision [118].

Various endogenous antioxidants, such as vitamins C and E, lutein, and zeaxanthin, are accumulated at the later stages of gestation, but they exist at low levels in preterm infants [120]. Moreover, the ischemic state restricts delivery of these antioxidants to the retina, further reducing the ability to scavenge retinal ROS. A study showed an elevation of the oxidative stress indicator, 8-hydroxy-2’-deoxyguanosine (8-OHdG) in ROP infants’ leukocytes and urine, implying that oxidative stress may play a substantial role in ROP pathogenesis, leading to DNA and cellular damages [121]. 

Until recently, the most popular means of ROP treatment are laser and cryotherapy, which involve the ablation of aberrant blood vessels in the peripheral retina. The alternative therapy of administering anti-VEGF drugs such as pegaptanib sodium, bevacizumab, ranibizumab, and aflibercept are being studied [122]. Ranibizumab was recently approved by the European Union for the treatment of ROP in September 2019 [123]. All these treatment strategies, despite their efficacy, are subjected to considerable limitations. Laser and cryotherapy may involve unintended and irreversible ocular tissue destruction, with partial loss of peripheral vision being a common side effect. Moreover, such invasive procedures require the use of general anesthesia, resulting in addition surgical risks to preterm infants. Anti-VEGF treatments, on the other hand, do not involve invasive tissue destruction and can be given with topical anesthetic. However, they may suppress the basal level of systemic VEGF, which is required for normal physiological developments for infants. Studies showed that deprivation of systemic VEGF activity is associated with complications like nephrotic syndrome, thromboembolic events, and delayed wound healing [124].

### 5.3. Lutein and ROP (Clinical Studies)

Earlier study indicated that lutein is effective in reducing oxidative stress in term newborns. Forty eight hours after birth, lutein supplementation could significantly reduce the infants’ level of hydroperoxides (TH), an oxidative marker, and raise their biological antioxidant potential (BAP) comparing with the control group [125]. Lutein, it has also been demonstrated, is well absorbed in preterm infants upon oral administration. There was a significant increase in lutein concentration by 13.5% and 16.7%, at 6 and 24 h after administration, respectively [126].

This evidence has raised researchers’ interest in investigating lutein’s effects on preterm infants with ROP (Table 9). A few studies showed that lutein may be able to improve ROP outcome in terms of its incidence and progression. ROP incidence in the lutein-treated group (43%) was lower than that in the control group (55%) [127] and proportion of late stage ROP was lower in lutein group (9.7%) compared with the control group (12.9%) [128]. Regarding the progression of ROP, there were fewer infants who developed severe ROP from mild ROP upon receiving supplementation containing lutein (8%) comparing with the control (28%) [129]. Manzoni et al. also found that the progression rate from early ROP to threshold ROP was lowered by around 50% with lutein intake [130]. However, it is worth noting that to date the number of aforementioned ROP studies related to lutein remains limited, and the findings of these studies were not statistically significant.

Failure to provide strong evidence concerning lutein’s effects on ROP may be due to several reasons: limited sample size, the nature of ROP as a multifactorial disease with many confounding risk factors, insufficient lutein dose administered, or simply a lack of sufficient clinical trials dedicated to this topic. Hence, more studies in larger scale are necessary should lutein be considered as one of the therapeutic options for preterm infants with ROP.

A recent review has also discussed the beneficial effects of lutein supplementation in pregnant and lactating women [131]. Carotenoid status in pregnant women can lower the risks of pregnancy pathologies and preterm birth that are results of elevated oxidative stress. Carotenoids are also among the few nutrients contained in breast milk, with its content dependent on mother’s dietary intake [131]. In fact, carotenoids, lutein and zeaxanthin in particular, are important in the development of the nervous system including the retina and the brain in newborn babies. Hence, lutein intake in both pregnant and lactating women may play an important role in ROP prevention by reducing risks of preterm birth and in neural development by supplementing newborns with adequate lutein. However, one should also be aware that commercially available carotenoid supplements may contain impurities, and their safety for intake during pregnancy has not been fully established. Moreover, it has been shown that the lutein and zeaxanthin contents in many powder filled lutein products may not comply with their label claim [132]. In view of this, it is recommended that pregnant women should ensure sufficient intake of lutein from normal diet rather than consuming high amounts of lutein supplements.

### 5.4. Lutein and ROP (Animal Studies)

Mouse or rat Oxygen-Induced Retinopathy (OIR) models are the most commonly used animal models to mimic human ROP and to study its progression [133,134]. These animal models are full-term neonates but are born with immature retinal development. Similar to preterm human infants, they are first exposed to a high oxygen environment for a few days to induce a central avascular zone and subsequently return to normal air to induce retinal neovascularization. Although the mouse model cannot fully represent the risk factors and pathologies in a preterm human baby with ROP, it is an easy and practical way to study ROP pathogenesis due to the availability of transgenic mice and the convenience in maintaining stable oxygen exposure [135].

Using the murine OIR model, lutein treatment was shown to promote normal retinal revascularization during the hypoxic stage by promoting endothelial tip cell formation and maintaining astrocytic template [136]. Nevertheless, the study was unable to demonstrate lutein’s effects in reducing retinal vasculature loss in initial hyperoxic phase, which is probably contributed by the insufficient intake of nutrients and maternal growth factors in the preterm infants. This may be a possible explanation why lutein appeared to be less effective in reducing ROP incidence in clinical trials. Due to its high bioavailability and ability to accelerate revascularization, lutein may be considered as a supplementary treatment for ROP in addition to other management procedures such as laser, cryotherapy and anti-VEGF injections.

## 6. Lutein Supplementation and Myopia

### 6.1. Myopia: Background

Myopia is considered to be one of the most prevalent eye disorders around the world, especially among Asian children. It is estimated that around 80–90% school students are affected by myopia in this region, among whom 10–20% suffer from high myopia [137]. Although myopia is not often considered as a serious eye disorder since vision can be easily corrected with glasses or contact lenses, it may increase the risk of other ocular pathologies such as glaucoma, retinal detachment, and lacquer cracks [138].

### 6.2. Myopia: Pathogenesis and Current Treatments

Under the condition of normal vision, parallel light rays from a distant object are converged by the lens and focus onto the retina where the photoreceptors are located. Diverging light rays from a near object are converged by the lens and focused behind the retina, and this is compensated by accommodation (ability of the lens to increase converging power), which brings the image forward onto the retina. In myopia, the light rays from a distant object are converged in front of the retina. Since the light rays are already overly converged, visual acuity cannot be compensated by accommodation and hence requires extrinsic corrections.

Axial length (AL) is the total length of the eyeball from the anterior surface of the cornea to the fovea, which is the sum of the anterior chamber depth, the lens thickness, and the vitreous chamber depth. A longer AL is associated with an increased risk and severity of myopia since light rays from distance object are focused in front of the retina. It has been shown that adults with low myopia (refractive error between −6 and 0 dpt) have an AL of around 24 mm while those with high myopia (refractive error more than −6 dpt) have an AL of around 30 mm [139]. It is also observed that AL generally increases rapidly early in life, followed by a mild increase during adulthood and a decrease in the elderly. This may be the explanation for the highest incidence and progression of myopia between childhood and early adulthood.

The most common management of myopia is by wearing eye glasses, in which light rays from distant objects are diverged by the corrective concave lens before entering the eyes so that they can focus sharply onto the retina. More invasive surgical treatments, such as Laser Assisted in situ Keratomileusis (LASIK) and Small Incision Lenticule Extraction (SMILE), are also available. In LASIK, a piece of corneal flap is cut using femtosecond laser, enabling the inner stromal bed to be trimmed with excimer laser based on the amount of refractive correction required before the flap is replaced. SMILE was later introduced as a refinement of the LASIK technique. In SMILE, femtosecond laser is used to cut out a piece of intrastromal lenticule, which is then removed through a small opening created by a minor incision. As SMILE does not involve the creation of corneal flap, it offers better preservation of the corneal biomechanical strength compared to LASIK [140].

### 6.3. Lutein and Myopia

To date, there is yet to be any large scale clinical trial aiming to directly investigate the association between lutein intake and incidence of myopia. However, a cross-sectional population study in 2017, which initially tried to demonstrate the effects of ultraviolet B radiation exposure and serum vitamin D on myopia, demonstrated an unexpected finding between lutein and myopia. It showed around 40% reduced odds of myopia (OR = 0.57) among subjects having the highest 20% of lutein concentration in plasma. The odds reduction was greater than that of ultraviolet B radiation exposure (OR = 0.81 in age 14–19; OR = 0.7 in age 20–39) and serum vitamin D concentrations, which showed no significant association [141].

There are other studies demonstrating lutein’s effects on improving myopia outcomes via mediation of other factors like MPOD and hyaluronic acid. A study showed that oral lutein supplementation significantly increased MPOD levels by 20% in myopic Japanese subjects (≤−4 dpt), opposed to zeaxanthin intake which resulted in no significant improvements in MPOD [142]. Another observational study indicated that a low level of MPOD may predispose myopic patients to lacquer cracks, which is one of the complications of high myopia characterized by the rupture of Bruch’s membrane and RPE [143].

Hyaluronic acid is a space-filling and water-retaining substance within the vitreous humor in the eyes, which plays a significant role in light refraction. The ability of hyaluronic acid to improve myopia-related outcomes was demonstrated by both animal and clinical studies. A guinea pig study showed that injectable biomimetic hyaluronic acid-based hydrogel could control progression of myopia without compromising retinal functions [144]. Another study in myopic patients who underwent keratectomy followed by prolonged topical steroid treatment found that they experienced myopic regression accompanied by suppressed normal formation of hyaluronic acid [145]. Moreover, carotenoids including lutein and zeaxanthin can act on retinoic acid receptors to induce HAS3-dependent hyaluronan synthesis in keratinocytes [146]. This evidence may provide clues concerning the benefits of lutein for myopic individuals due to its ability in promoting hyaluronic acid synthesis, which is shown to be protective against myopia.

Furthermore, one observational study investigating Chinese adult population had concluded a significant inverse association between MPOD and AL. A longer AL, which is a major predictor of myopia occurrence and severity, was observed in subjects with lower levels of macular pigments including lutein and zeaxanthin [147]. The investigators of this study proposed a possible explanation for this observation, which involves central retinal thickness. They reviewed other published observational studies and concluded a positive correlation between central retinal thickness and MPOD and a significant negative relationship between central retinal thickness and AL [148,149,150]. Hence, a myopic individual with longer AL has thinner central retina and lower MPOD. Although some researchers were skeptical about this observation due to the study’s limited sample size (173 myopic subjects) and insufficient efforts to address confounding factors like age and dietary carotenoids intake [151], it still provided an important basis for further investigations concerning the correlation between lutein concentration in eye and myopia. 

## 7. Lutein Supplementation and Cataract

### 7.1. Cataract: Background and Treatment

Cataract is one of the most common age-related eye diseases. Affecting an estimated population of 95 million worldwide [152], it contributes to nearly 90% of blindness in developed countries [153]. It is characterized by the opacification or clouding of lens, which reduces the amount of light passing through to reach the retina, resulting in a blurred vision. Cataract is a multifactorial disease, but aging is considered to be the single commonest risk factor for cataract development. It is estimated that over 90% of population aged 70 or above present some evidence of cataract with varying severity [154]. 

Human lens is a crystalline substance made up of a single layer of cuboidal epithelium surrounded by a lens capsule. It consists of mainly water and protein, with the main function of providing clear passage for light [155]. New cells are continuously formed in the lens. They differentiate into lens fibers by stretching and losing their organelles to maintain lens transparency [153]. Upon lens aging, as more fibers are deposited, the lens nucleus (central core of the lens) hardens and becomes more compacted, occasionally accompanied with other senile changes such as yellowish or greyish discoloration. This is known as nuclear sclerosis, which increases lens opacity and disrupts normal passage of light, resulting in cataract [156]. The fact that cells in the lens are devoid of organelles makes them more susceptible to photo-oxidative insults, leading to breakdown and aggregation of proteins, which also contribute to lens discoloration and opacity [153]. Many more risk factors that predispose one to cataract have been identified, which include smoking, hypertension, diabetes, ocular trauma, steroid use, hypocalcemia, hypothyroidism, severe dehydration, nutritional deficiencies, etc. [155], while the precise pathophysiological mechanisms of how each factor can contribute to reduced lens opacity remain to be thoroughly studied and elucidated.

To date, surgery remains the only means of medical treatment that can effectively reverse vision loss caused by cataract. Three main surgical techniques are currently developed, which include intracapsular cataract extraction, extracapsular cataract extraction, and phacoemulsification. Among the three, phacoemulsification is most commonly used nowadays since it is least invasive and has the lowest risk of complications. It involves emulsification of the lens nucleus by an ultrasonic probe and its replacement by a synthetic intraocular lens [153].

### 7.2. Lutein and Cataract

Situated in the front of the eye, the lens is constantly subjected to oxidative stress by UV-light and other sources. A number of antioxidant defense systems including antioxidant enzymes such as superoxide dismutase and antioxidants are utilized in the lens to protect against oxidative stress-mediated damages [157,158]. Glutathione is the principal antioxidant in the lens. Reduced glutathione (GSH) is present at very high concentration in the young lens, yet the level of glutathione drops significantly with age, particularly in the central region of the lens. This results in increased oxidative stress, later lens protein aggregation, and ultimately lens opacification and cataract [153]. As cataract is associated with oxidative stress, antioxidant supplementation has been considered in the treatment of cataract. Another antioxidant present in the lens is lutein. Being a blue-light filter, a potent antioxidant and a dietary supplement, lutein’s possible role in cataract management has been of interest to many researchers [22].

Currently, the studies concerning lutein’s effect on cataract have been relatively controversial. The Beaver Dam Eye Study showed that subjects with the highest quintile of lutein intake had 50% reduction in likelihood of cataract incidence compared with those in the lowest quintile [159]. Another study among the Finnish elderly population also reported similar results, in which subjects with the highest tertile of plasma lutein concentration had 42% lower risk of developing nuclear cataract as compared with those in the lowest tertile [160]. However, one study compared the carotenoid levels between normal lenses and caractous lenses of the American population and discovered that there were no significant differences in lutein and zeaxanthin contents [161]. Another study demonstrated that despite lutein’s ability in reducing oxidative damage, its effect cannot compensate for the glutathione depletion in age-related cataract [162]. In the recent AREDS2 study, it was concluded that the evidence of daily supplementation of lutein in reducing risks of cataract surgery or vision loss was not statistically significant. However, lutein supplementation was able to reduce surgery risks among subjects having the lowest quintile of dietary lutein intake [163]. This finding agrees with the pre-existing knowledge that nutritional deficiency is one of the risk factors for cataract, suggesting that the beneficial role of lutein is most prominently observed among the undernourished population.

## 8. Conclusions

Lutein is a xanthophyll primarily synthesized in plants but not in humans. It is highly concentrated in the macula and exhibits various features such as anti-inflammatory, anti-oxidative, and blue light-filtering effects. Due to these eye-protective properties and its relatively high safety profile, lutein is often considered by many researchers to be a potential therapeutic alternative/adjunct for various kinds of eye diseases. To date, most efforts are devoted to investigating lutein’s effects on AMD and DR, since they are the most common causes of visual impairment in the working and aging population with oxidative damages and inflammation involved in their pathological processes. Most animal and clinical studies have provided promising results, supporting lutein’s efficacy in delaying development and progression of these conditions. At the same time, more evidence has emerged to demonstrate lutein’s possible role in alleviating outcomes of other eye diseases including ROP, myopia, and cataract. However, clinical trials with larger sample size are required to strengthen these associations and to fully evaluate lutein’s protective roles. Moreover, comprehensive understanding of the optimal dose of lutein for different age and gender, the long-term safety of lutein, and physiological interactions with other drugs and supplements is still required should lutein be considered a pharmacological adjunct therapy for these eye diseases in the future. 

## Figures and Tables

**Table 1:** Common dietary sources of xanthophylls [5].

Food Items	Lutein (μg/g Fresh Weight)	Zeaxanthin (μg/g Fresh Weight)
Vegetables		
Basil	70.5	
Kale	48.0–114.7	
Leek	36.8	
Parsley	64.0–106.5	
Red pepper	2.5–85.1	5.9–13.5
Egg		
Egg yolk	3.8–13.2	
Nuts		
Pistachio	7.7–49.0	
Grains		
Corn	21.9	10.3
Einkorn wheat	7.4	0.9
Khorasan wheat	5.5	0.7
Durum wheat	5.4	0.5

**Table 2:** Basic clinical classification of age-related macular degeneration (AMD).

Basic Clinical Classification [59,60]			
Stage	Drusen	Pigment Abnormalities	Additional Features
No aging changes	Absent	Absent	Nil
Normal aging changes	Small (≤63 μm)	Absent	Nil
Early AMD	Medium (>63 μm but ≤125 μm)	Absent	Nil
Intermediate AMD	Large (>125 μm)	Present	Nil
Late AMD	Large (>125 μm)	Present	Neovascular AMD/geographic atrophy

**Table 3:** AREDS categorization of AMD.

Age-Related Eye Disease Study (AREDS) Classification [61]	
**Category**	
1	No drusen/Small, non-extensive drusen in both eyes
2	Small, extensive drusen/Intermediate, non-extensive drusen/Pigment abnormalities in at least one eye
3	Intermediate extensive drusen/Large drusen/Noncentral geographic atrophy in at least one eye
4	Advanced age-related macular degeneration defined by geographic atrophy, retinal pigment epithelial detachment in one eye, choroidal neovascularization or scars of confluent photocoagulation/Visual acuity less than 20/32 induced by lesions like large drusen in the fovea in only one eye due to nonadvanced age-related macular degeneration

**Table 4:** Studies indicating protective effects of lutein for AMD.

	Name	Study Design	Subject	Results
Arch Ophthalmol 1993 [76]	Eye Disease Case-Control Study (EDCCS)	Case control study	421 AMD patients, 615 controls	High serum lutein level reduces neovascular AMD risk
Richer 2004 [84]	Lutein Antioxidant Supplementation Trial (LAST)	Randomized control trial, 12-month follow up	90 Atrophic AMD patients in USA	Lutein supplements improve visual function
Richer 2007 [78]	Lutein Antioxidant Supplementation Trial II (LASTII)	Randomized control trial, 12-month follow up	90 Atrophic AMD patients in USA	Lutein increases macular pigment optical density (MPOD)
Tan 2008 [75]	The Blue Mountains Eye Study (BMES)	Population based cohort study, follow up after 5 and 10 years	2454 Australians aged ≥49	High lutein intake reduces long-term AMD risk
Neelam 2008 [85]	Carotenoids and co-antioxidants in age-related maculopathy (CARMA) study	Randomized control trial, 12-month follow up	433 Caucasian AMD patients aged ≥55	Lutein increases both macular pigment level and visual acuity
Ho 2011 [86]	The Rotterdam Study	Nested case-control study, mean 8.6 year follow up	2167 individuals aged ≥55 with genetic variants CFH Y402H and LOC387715 A69S	High lutein intake reduces early AMD risk in those at high genetic risk
Weigert 2011 [79]	Lutein Intervention Study Austria (LISA)	Randomized control trial, 6-month follow up	126 AMD patients	Lutein increases MPOD
Age-Related Eye Disease Study 2 Research Group 2013 [50]	Age-Related Eye Disease Study 2 (AREDS2)	Randomized control trial	4203 intermediate or advanced AMD patients aged 50 to 85	AREDS2 formula containing lutein reduces progression to advanced AMD
Murray 2013 [80]	Combination of Lutein Effects in the Aging Retina (CLEAR) study	Randomized control trial, 12- month duration	72 patients, mean age of 70.5	Lutein increases MPOD and slows down visual acuity reduction

**Table 5:** Studies showing no association between lutein and AMD development.

	Name	Study Design	Subject	Results
VandenLangenberg 1998 [82]	Beaver Dam Study	Population based cohort study, 5-year incidence	1709 adults in USA	Too few incidence, unable to show association of lutein with age-related maculopathy
Moeller 2006 [83]	Carotenoids in Age-related Eye Disease Study (CAREDS)	Population based ancillary study, 6 years prevalence	1787 women, aged 50–79	Lowered odds ratio of intermediate AMD only in age group >75
Cho 2008 [87]	Nurses’ Health Study, Health Professionals Follow-up Study	Prospective follow-up study	77562 women 40,866 men, aged ≥50	Lutein intake not strongly related to age-related maculopathy
Cho 2008 [81]	Nurses’ Health Study, Health Professionals Follow-up Study	Prospective follow-up study	71494 women and 41,564 men, aged ≥50	Lutein has no protective role against early AMD

**Table 6:** International Clinical Disease Severity Scale for DR [94,95].

DR Severity Scale	Characteristics
No apparent retinopathy	No recognizable diabetic fundus changes
Mild Non-proliferative diabetic retinopathy (NPDR)	Presence of at least one microaneurysm
Moderate NPDR	Presence of microaneurysms, intraretinal hemorrhages or venous beading
Severe NPDR	Presence of hemorrhages in all 4 fundus quadrants, venous beading in at least 2 quadrants, or intraretinal microvascular abnormalities (IRMA)
PDR	Presence of neovascularization of the disc, the retina, the iris, or the angle, or presence of vitreous hemorrhage or tractional retinal detachment

**Table 7:** Studies showing the association between MPOD and diabetic retinopathy (DR) risks.

	Study Design	Subject	Results
Davies 2002 [104]	Case control study	30 non-diabetic subjects 26 diabetic subjects	MPOD is lower in diabetic subjects compared to non-diabetic group. MPOD is also lower in diabetic patients with DR compared to diabetic patients without
Lima 2010 [105]	Case control study	14 non-diabetic subjects 17 diabetic subjects without DR 12 diabetic subjects with NPDR	MPOD is lower in type 2 diabetic subjects (with or without DR) compared to non-diabetic group

**Table 8:** Studies showing the association between blood lutein level and DR risks.

	Study Design	Subject	Results
Brazionis 2009 [106]	Cross-sectional study	78 diabetic subjects without DR 33 diabetic subjects with NPDR	Plasma level of combined lutein and zeaxanthin is lower in patients with NPDR than those without NPDR
Hu 2011 [107]	Interventional study	30 non-diabetic subjects 30 NPDR subjects with lutein supplement 30 NPDR subjects without lutein supplement	Administration of lutein and zeaxanthin increase their plasma levels and improve visual acuity and contrast sensitivity in NPDR
Zhang 2017 [42]	Randomized control trial	31 NPDR subjects randomized into lutein and placebo group	Administration of lutein improves contrast sensitivity in NPDR patients

**Table 9:** Studies showing the association between lutein supplementation and Retinopathy of prematurity (ROP) outcomes.

	Subjects	Treatment	Results
Romagnoli 2011 [128]	Preterm infants <33 week gestational age 31 treatment group 32 control group	lutein (0.5 mg/kg), zeaxanthin (0.02 mg/kg)	Lutein did not lead to significance difference in ROP incidence
Rubin 2012 [129]	Preterm infants <33 week gestational age 92 treatment group 91 control group	lutein/zeaxanthin, lycopene and β-carotene (24 kcal/oz in hospital, 22 kcal/oz post-discharge)	Supplementation raised plasma lutein level and rod photoreceptor sensitivity. No significance difference in ROP incidence
Dani 2012 [127]	Preterm infants <33 week gestational age 58 treatment group 56 control group	lutein (0.14 mg), zeaxanthin (0.006 mg)	Lutein did not affect outcome of ROP
Manzoni 2013 [130]	229 preterm infants <32 week gestational age	lutein (0.14 mg), zeaxanthin (0.0006 mg)	Lutein did not lead to significance difference in ROP outcome

## Abbreviations

AGEs	Advanced glycation end products
AL	Axial length
AMD	Age-related macular degeneration
BDNF	Brain-derived neurotrophic factor
COX-2	Cyclooxygenase-2
CRN	Council for Responsible Nutrition
DR	Diabetic retinopathy
ERG	Electroretinogram
ERK	Extracellular signal-regulated kinase
FDA	US Food and Drug Administration
GGPP	Geranylgeranyl pyrophosphate
GPx	Glutathione peroxidase
GRAS	Generally Regarded as Safe
GSH	Glutathione
HbA1c	Hemoglobin A1c
HDL	High-density lipoproteins
IGF-1	Insulin-like growth factor-1
IL-1β	Interleukin 1β
iNOS	Inducible nitric oxide synthase
IRMA	Intraretinal microvascular abnormalities
LASIK	Laser Assisted in situ Keratomileusis
LDL	Low-density lipoproteins
mfERG	Multifocal electroretinography
MPOD	Macular pigment optical density
NF-κB	Nuclear factor-kappa B
NPDR	Non-proliferative diabetic retinopathy
OCT	Optical coherence tomography
OIR	Oxygen-Induced Retinopathy
PDR	Proliferative diabetic retinopathy
ROP	Retinopathy of prematurity
ROS	Reactive oxygen species
RPE	Retinal pigment epithelium
SMILE	Small Incision Lenticule Extraction
VLDL	Very-low-density lipoproteins
